# Supplementary material for: The Pitfall of White Blood Cell Cystine Measurement to Diagnose Juvenile Cystinosis
Source: Int J Mol Sci. 2023 Jan 9;24(2):1253. doi: 10.3390/ijms24021253 (PMC9864853; doi:10.3390/ijms24021253)
Supplement: Supplementary file 1 [file ijms-24-01253-s001.zip › Supplementary Table S1.pdf]

Clinical laboratory values (blood and fibroblasts)

	nmol 1/2 cystine/mg protein
c.530 A>G WBC	1.31
c.530A>G fibroblasts	3.4

c.530 A>G ciPTEC and ciPODO measurements

	Solution	Total Volume NEM+SSA (µl)	Protein µg/mL	Cys µM	Cys nmol	Cys nmol/mg protein	1/2 cystine nmol/mg protein	average	stdev
c.530 A>G PTECs	5mM NEM+12%SSA	100+50	563.8	4.02	4018.304448	7.13	14.25	13.39	0.69
c.530 A>G PTECs	5mM NEM+12%SSA	100+50	806.1	5.04	5035.517598	6.25	12.49		
c.530 A>G PTECs	5mM NEM+12%SSA	100+50	752.4	5.13	5129.770467	6.82	13.64		
c.530 A>G PTECs	5mM NEM+12%SSA	100+50	776.4	4.90	4903.241845	6.32	12.63		
c.530 A>G PTECs	5mM NEM+12%SSA	100+50	811.1	5.49	5486.540248	6.76	13.53		
c.530 A>G PTECs	5mM NEM+12%SSA	100+50	728.9	5.04	5035.963029	6.91	13.82		
c.530 A>G PODOs	5mM NEM+12%SSA	100+50	706.3	2.73	2733.833538	3.87	7.74	8.59	0.62
c.530 A>G PODOs	5mM NEM+12%SSA	100+50	625.8	2.72	2719.969076	4.35	8.69		
c.530 A>G PODOs	5mM NEM+12%SSA	100+50	659.9	3.10	3099.073063	4.70	9.39		
c.530 A>G PODOs	5mM NEM+12%SSA	100+50	766.6	3.46	3462.062907	4.52	9.03		
c.530 A>G PODOs	5mM NEM+12%SSA	100+50	756.2	3.03	3026.392672	4.00	8.00		
c.530 A>G PODOs	5mM NEM+12%SSA	100+50	707.3	3.07	3072.633352	4.34	8.69		

Control values

	Solution	Total Volume NEM+SSA (µl)	Protein µg/mL	Cys µM	Cys nmol	Cys nmol/mg protein	1/2 cystine nmol/mg protein	average	stdev
Control PTECs	5mM NEM+12%SSA	100+50	797.1	1.15	1152.108162	1.45	2.89	2.04	0.68
Control PTECs	5mM NEM+12%SSA	100+50	751.8	0.73	725.3667974	0.96	1.93		
Control PTECs	5mM NEM+12%SSA	100+50	778.1	0.81	812.0816146	1.04	2.09		
Control PTECs	5mM NEM+12%SSA	100+50	797.1	0.49	494.496549	0.62	1.24		
Control PODOs	5mM NEM+12%SSA	200+100	297.6	0.63	626.5411046	2.11	4.21	5.01	1.65
Control PODOs	5mM NEM+12%SSA	200+100	212.2	0.73	732.9975204	3.45	6.91		
Control PODOs	5mM NEM+12%SSA	200+100	319.5	0.62	623.6802777	1.95	3.90		
